# Delayed Ileal Hemorrhage After Blunt Abdominal Trauma Successfully Managed With Capsule Endoscopy: A Case Report

**DOI:** 10.7759/cureus.93276

**Published:** 2025-09-26

**Authors:** Shimpei Asada, Naoki Kawahara, Koji Morishita, Shusuke Mori

**Affiliations:** 1 Department of Emergency Medicine, Tokyo Women's Medical University, Tokyo, JPN; 2 Trauma and Acute Critical Care Center, Institute of Science Tokyo, Tokyo, JPN

**Keywords:** abdominal trauma, capsule endoscopy, gastrointestinal bleeding, ileal hemorrhage, mesenteric injury, nonoperative management, patency capsule, small bowel injury

## Abstract

Blunt abdominal trauma typically results in solid organ injury, while hollow viscus injury is rare but associated with high morbidity when diagnosis is delayed. Delayed complications most often manifest as stenosis, necrosis, or, less commonly, perforation, which, although typically an early complication, has also been reported in delayed settings; moreover, delayed intraluminal hemorrhage is exceptionally uncommon. We present the case of an 84-year-old man who sustained blunt abdominal trauma after a fall. Initial computed tomography demonstrated a perihepatic hematoma and segmental ileal wall edema without extravasation, and conservative management was initiated. Hematochezia occurred on hospital days 3 and 16, with angiography confirming active bleeding from a superior mesenteric artery branch, successfully treated with coil embolization. Despite transfusion of 16 units of red blood cells, recurrent bleeding continued until day 26, though subsequent imaging revealed no extravasation. To further evaluate, a patency capsule was administered, confirming luminal patency, followed by capsule endoscopy on day 36. Capsule images revealed multiple healing ileal ulcers without active bleeding or stenosis. Based on these findings, conservative management was continued, and the patient was discharged on day 70 without recurrence. This case highlights the diagnostic and therapeutic challenges of delayed post-traumatic small bowel bleeding, a rare but clinically significant condition. Conventional imaging and angiography often fail to distinguish healing from ongoing hemorrhage, leaving management decisions uncertain. Capsule endoscopy, safely preceded by patency testing, provided definitive mucosal assessment, clarified the etiology, and guided nonoperative management, thereby avoiding unnecessary laparotomy. Capsule endoscopy should be considered a valuable adjunct in trauma care for stable patients with unexplained recurrent bleeding, as it has the potential to refine diagnostic algorithms and reduce morbidity.

## Introduction

Blunt abdominal trauma is a major contributor to morbidity and mortality in trauma patients, with hollow viscus injury representing one of the most diagnostically challenging entities. Hollow viscus injury occurs in fewer than 2% of blunt trauma admissions but carries disproportionately high mortality compared with solid organ injury, with reported mortality rates as high as 15-20% [1]. In elderly patients, outcomes are further worsened by frailty, comorbidities, and limited physiologic reserve, making timely recognition especially critical [2]. Although stenosis, obstruction, and ischemia are recognized as delayed complications, intraluminal bleeding presenting weeks after trauma is exceedingly rare. This rarity contributes to diagnostic uncertainty, as conventional imaging and angiography may not be able to adequately distinguish active hemorrhage from mucosal healing. In this context, capsule endoscopy, after confirming luminal patency, may provide direct visualization of mucosal healing and support safe continuation of conservative management [3-8].

Outcomes are strongly influenced by diagnostic timing, as delays increase the risk of sepsis and death. While differential diagnoses such as inflammatory bowel disease or ischemic enteritis may mimic post-traumatic changes, delayed manifestations of small bowel trauma, including stenosis, obstruction, and ischemia, are well documented. Clinically significant sequelae have been described in both pediatric and adult cohorts. In contrast, delayed intraluminal bleeding is exceedingly rare, creating diagnostic and therapeutic uncertainty that justifies careful case reporting [9].

Despite advances in multidetector computed tomography (CT), diagnostic performance for bowel and mesenteric injury remains imperfect. CT with intravenous contrast alone can achieve a sensitivity of up to 95% and a specificity of 99%, but subtle mucosal ischemia or mesenteric contusions may still evade detection [10]. In elderly patients, blunt abdominal trauma is associated with greater morbidity and mortality due to frailty, comorbidities, and reduced physiological reserve [11]. By contrast, in pediatric cohorts, small bowel injury is uncommon, affecting approximately 5% of admissions, and recognition is frequently delayed despite careful monitoring [12].

Beyond acute perforation, small bowel trauma can result in delayed complications such as stenosis, obstruction, or ischemia, which may present days to months after injury [3-6]. Several case reports and experimental studies have highlighted the potential for mesenteric contusions and submucosal hematomas to evolve into clinically significant sequelae such as obstruction, stenosis, or ischemia [13]. These delayed outcomes underscore the importance of long-term vigilance in trauma patients.

In contrast to delayed complications such as stenosis or ischemia, delayed intraluminal bleeding due to mesenteric arterial injury is exceedingly rare. However, case reports have described superior mesenteric artery branch hemorrhage following blunt trauma [14-16]. Conventional imaging and angiography may not adequately distinguish active hemorrhage from healing mucosa, leaving clinical decisions uncertain [7,10]. Capsule endoscopy, initially developed for the diagnosis of obscure gastrointestinal bleeding, has proven superior to push enteroscopy and small bowel radiography, with diagnostic yields exceeding 60% [7]. Modern cohorts confirm high yields in overt bleeding and substantial impact on clinical management [17]. Concerns regarding capsule retention have been addressed through the introduction of the patency capsule, which allows for confirmation of luminal patency before diagnostic capsule endoscopy is attempted [8,18]. Systematic reviews demonstrate that overall retention rates are low, approximately 2%, and substantially lower when patency testing is performed [19,20].

Here, we describe an elderly patient with delayed ileal hemorrhage after blunt trauma, in whom capsule endoscopy clarified the diagnosis and enabled safe conservative management.

## Case presentation

An 84-year-old man with a history of hypertension sustained blunt abdominal trauma after falling down nine stairs at 7 a.m. He was initially evaluated at a local hospital and subsequently transferred to our institution by 7 p.m. on the same day. He presented as alert and hemodynamically stable, with a blood pressure of 128/72 mmHg, a heart rate of 82 beats per minute, a respiratory rate of 18 breaths per minute, an oxygen saturation of 98% on room air, and a temperature of 36.7°C, accompanied by localized tenderness in the periumbilical area. His past medical history was limited to hypertension, and although inflammatory markers were elevated, there were no clinical or radiographic findings suggestive of infection at admission. Laboratory studies on admission are summarized in Table 1.

**Table 1:** Laboratory findings on admission WBC: white blood cell count, RBC: red blood cell count, AST: aspartate aminotransferase, ALT: alanine aminotransferase, LDH: lactate dehydrogenase, γ-GTP: gamma-glutamyl transpeptidase, amylase: serum amylase, BUN: blood urea nitrogen, CRP: C-reactive protein, PT: prothrombin time, INR: international normalized ratio, APTT: activated partial thromboplastin time

Test item	Measured value	Unit	Reference range
WBC	7,900	/µL	4,000-9,000
RBC	4.17	×10^6^/µL	4.2-5.5
Hemoglobin	13.3	g/dL	13.5-18.0
Hematocrit	41.3	%	39-52
Platelet	221	×10^3^/µL	150-350
AST	32	U/L	13-33
ALT	17	U/L	8-42
LDH	314	U/L	119-229
γ-GTP	10	U/L	10-47
Amylase	79	U/L	44-132
Total bilirubin	1.6	mg/dL	0.3-1.2
BUN	61.1	mg/dL	8-20
Creatinine	1.96	mg/dL	0.6-1.1
CRP	5.97	mg/dL	0.0-0.3
PT (INR)	0.96	-	0.90-1.14
APTT	24.7	sec	24-40
Fibrinogen	404	mg/dL	150-340
D-dimer	49.1	µg/mL	0-1.0

**Figure 1:**
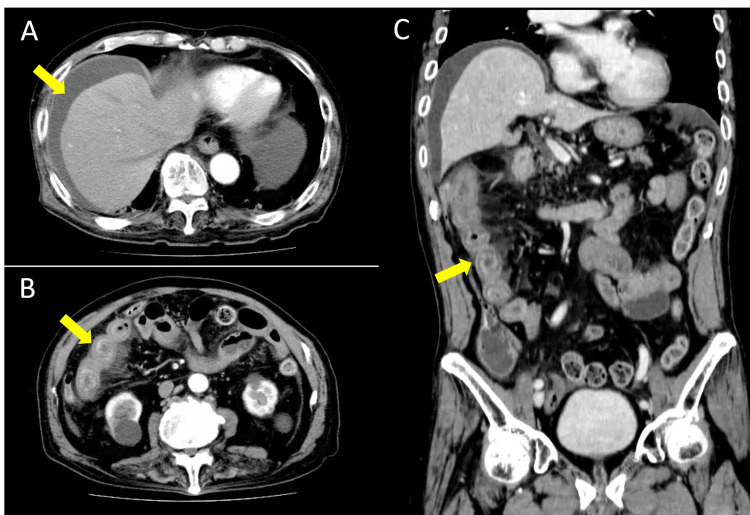
Initial contrast-enhanced CT performed on admission (arterial phase) A: The axial view of the liver demonstrates a low-grade hepatic injury with a perihepatic hematoma (arrow), with measured attenuation values of 30–35 Hounsfield units, consistent with acute hemorrhage. B: The axial section through the right upper abdomen shows marked wall thickening and edema of the small intestine (arrow). C: The coronal reconstruction further confirms segmental ileal wall edema (arrow). These findings indicated blunt abdominal trauma with mesenteric contusion but no active bleeding, supporting the decision for initial nonoperative management. CT: computed tomography

Initial contrast-enhanced CT revealed a perihepatic hematoma consistent with low-grade hepatic injury and segmental ileal wall edema with mesenteric stranding. Notably, pronounced bowel wall thickening and edema were observed in the right upper abdominal small intestine, but no active extravasation was identified (Figure 1).

On hospital day 3, the patient developed hematochezia. Repeat CT demonstrated progression of ileal wall thickening and mesenteric edema, though mucosal enhancement remained preserved. Conservative therapy was continued with bowel rest, parenteral nutrition, and serial clinical and laboratory monitoring. Between hospital days 4 and 15, the patient remained hemodynamically stable, with no further hematochezia. He was managed conservatively with bowel rest and parenteral nutrition, later transitioning to limited oral intake. No new abdominal findings were noted during this period.

**Figure 2:**
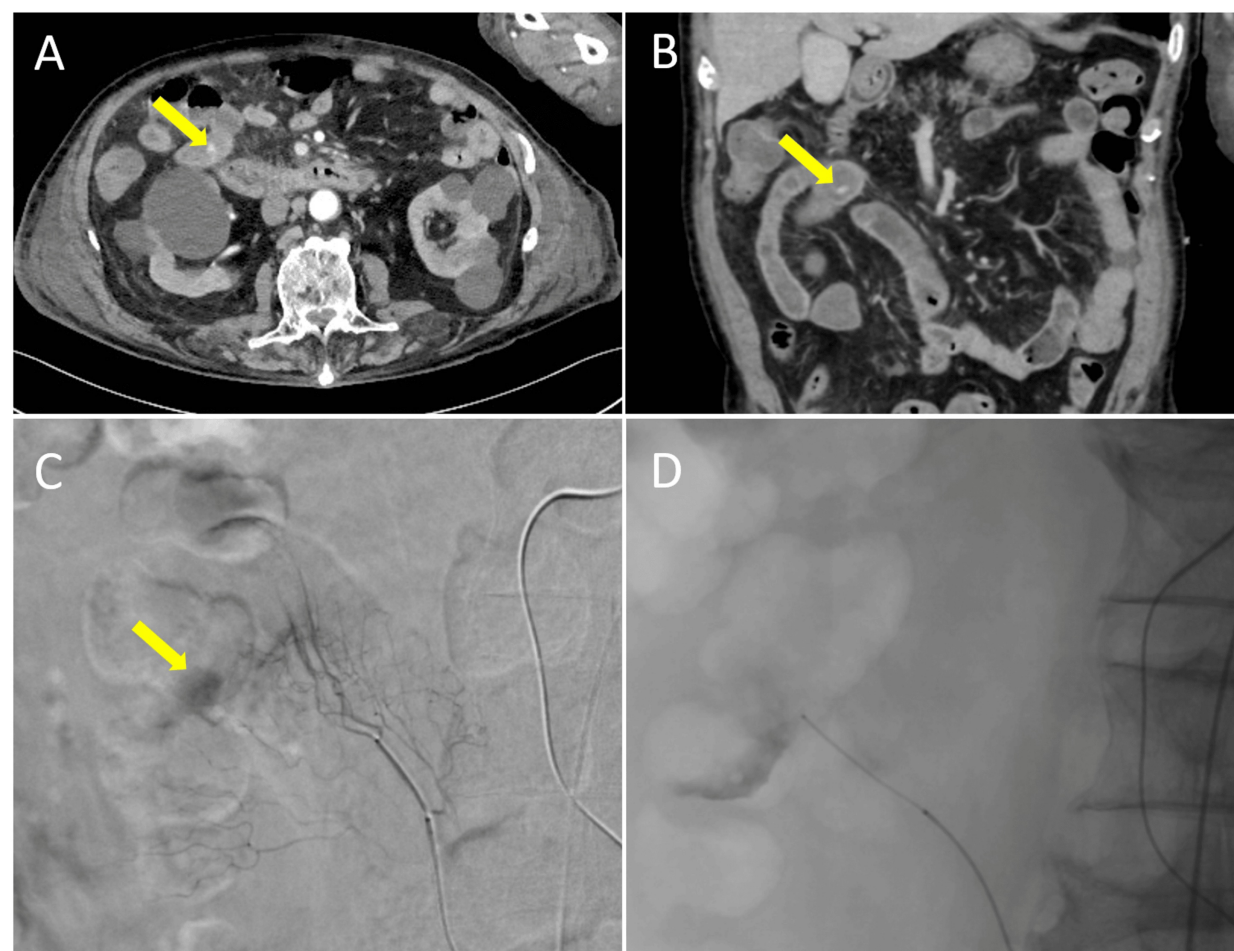
CT angiography in the arterial phase obtained on hospital day 16 following recurrent hematochezia A, B: Both axial (A) and coronal (B) views demonstrate active contrast extravasation from the ileum, localizing the source of bleeding (arrows). These images were critical in identifying delayed post-traumatic hemorrhage and prompted urgent interventional radiology. C: Mesenteric angiography performed after CT angiography confirmation. A selective study of a superior mesenteric artery branch revealed active extravasation (arrow). D: Super-selective coil embolization was subsequently performed, resulting in temporary hemostasis. This step illustrates the effectiveness of interventional radiology in stabilizing traumatic bleeding, even though further episodes of hematochezia occurred later. CT: computed tomography

On day 16, massive hematochezia recurred with hypotension, and hemoglobin fell to 5.9 g/dL, necessitating urgent transfusion and resuscitation. CTA revealed active extravasation in the ileum (Figure 2A-2B); mesenteric angiography confirmed bleeding from a superior mesenteric artery branch. Selective and super-selective coil embolization achieved temporary hemostasis (Figure 2C-2D). Despite this intervention, intermittent hematochezia persisted, requiring transfusion of a total of 16 units of red blood cells by day 20, including 12 units administered after embolization due to persistent bleeding.

**Figure 3:**
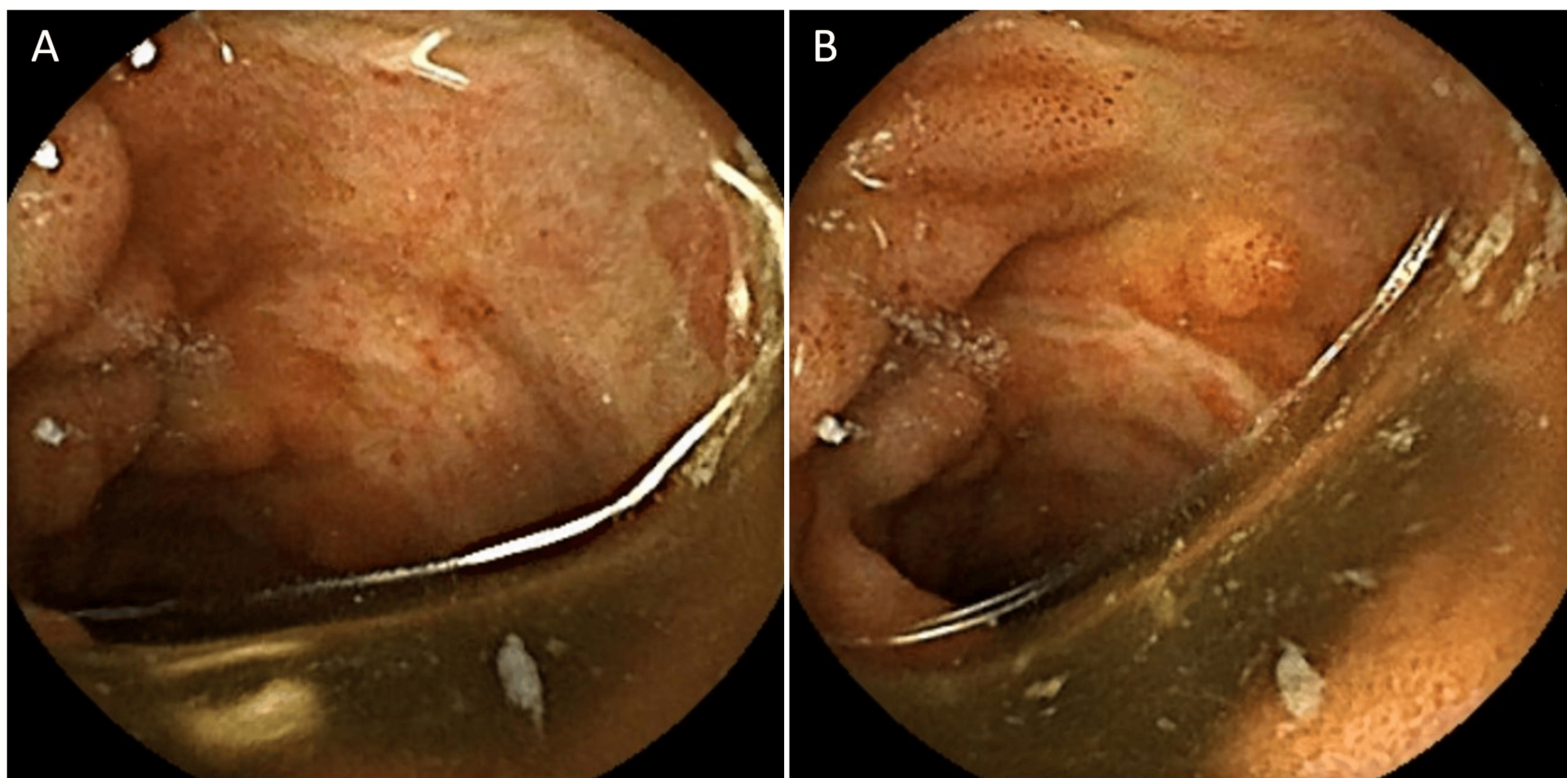
Capsule endoscopy performed on hospital day 36 after confirmed passage of a patency capsule A, B: The images show multiple healing ulcers in the mid-ileum with fibrinous exudates, without evidence of active bleeding, thereby excluding ongoing hemorrhage, and also without stenosis or obstruction. These findings clarified the etiology of recurrent hemorrhage and confirmed mucosal healing, allowing safe continuation of conservative management without laparotomy.

On day 26, recurrent bleeding occurred without radiographic extravasation. Exploratory laparotomy was considered; however, to evaluate for stricture or mucosal pathology, a patency capsule was administered orally at noon on hospital day 26, and expulsion was confirmed by abdominal radiograph 18 hours later. Capsule endoscopy was then performed on day 36, revealing multiple healing ulcers in the mid-ileum with fibrinous exudates but no active bleeding or stenosis (Figure 3).

Following this, conservative management was continued. The patient stabilized after bowel rest and parenteral nutrition, later resumed oral intake, and was discharged on day 70 without recurrence. A detailed chronological summary of the patient’s hospital course is provided in Table 2, which highlights the timing of trauma, interventions, rebleeding episodes, transfusion requirements, capsule procedures, and discharge.

**Table 2:** Clinical timeline and key management milestones NPO: nil per os, IV: intravenous, TPN: total parenteral nutrition, EN: enteral nutrition, PRBC: packed red blood cells, NOM: nonoperative management, CTA: computed tomography angiography

Hospital day	Event/imaging	Hb (g/dL)	PRBC units (cumulative)	Nutrition	Notes
0	Fall; CT: low-grade hepatic injury, ileal edema	13.3	-	NPO	Admitted for NOM
3	Hematochezia; CT: edema↑, no extravasation	10.6	-	NPO	Conservative treatment
5	-	9.5	-	NPO + TPN	-
16	Massive hematochezia; arterial-phase CTA: active extravasation → SMA branch embolization	5.9	6U	EN → TPN	Selective → super-selective coil
17	-	5.8	6U	-	-
18	-	7.8	-	NPO + TPN	-
19	Intermittent bleeding persists	7.2	4U	NPO + TPN	-
20	Stop hematochezia	9.2	-	NPO + TPN	Post-embolization
21	Enteral nutrition was initiated	8.4	-	EN	-
26	Oral intake resumed	7.9	-	Oral intake resumed	-
27	Recurrent bleeding; CT: no extravasation	6.4	4U	NPO + TPN	Laparotomy considered
28	-	7.3	2U	NPO + TPN	-
30	-	7.4	2U	NPO + TPN	-
	Patency capsule passed	-	-	NPO + TPN	-
36	Capsule endoscopy was administered orally	7.7	-	NPO + TPN	-
37	The capsule endoscope reached the colon: healing ileal ulcers, no bleeding/stricture	7.8	-	NPO + TPN	Gastric transit time 2 h 25 m, small intestine transit time 3 h 6 m
39	Oral intake resumed	7.5	-	Oral intake resumed	-
42	Onset of mild cholecystitis	8	-	NPO + TPN	-
49	Oral intake resumed	8.1	-	Oral intake resumed	-
60	Onset of urinary tract infection	-	-	-	-
70	Discharge	-	-	Full diet	No recurrence

## Discussion

This case underscores both the rarity and clinical significance of delayed post-traumatic small bowel hemorrhage. Arterial injury associated with blunt abdominal trauma is rare but accounts for fewer than 2% of admissions, yet carries mortality rates as high as 15-20%, with several reports highlighting isolated superior mesenteric artery branch injury requiring intervention [14-16]. Diagnostic delays remain a significant contributor to adverse outcomes; however, even state-of-the-art CT scans cannot reliably detect subtle or evolving injuries [10,21]. While delayed recognition of small bowel injury has been reported in pediatric cohorts [12], geriatric trauma patients, such as in the present case, are particularly vulnerable to delayed diagnosis and adverse outcomes, with studies showing significantly higher mortality and risk of missed injuries in the elderly [2].

The delayed sequelae of blunt bowel trauma demonstrate a heterogeneous natural history. Ileal stenosis has been observed months after injury [3], strictures have required laparoscopic resection [6], and late obstruction has been linked to mesenteric vascular compromise [4]. Even minor mesenteric injuries may progress to ischemia [5]. Experimental evidence supports these clinical observations, showing that contusion size and ischemia are predictors of delayed necrosis or stricture formation [13]. Collectively, these studies emphasize that the spectrum of delayed intestinal injury is broad and often unpredictable.

Unlike stenosis or ischemia, delayed intraluminal bleeding has been documented only rarely, with reported incidence limited to case reports and small series describing superior mesenteric artery branch hemorrhage after blunt trauma [14-16]. While alternative explanations such as Crohn’s disease or infectious enteritis could be considered in a patient with elevated inflammatory markers and ascites, the absence of a prior gastrointestinal history, the temporal relationship to trauma, the CT findings of mesenteric contusion, and the capsule endoscopy showing healing traumatic ulcers without chronic features strongly support trauma as the underlying etiology in this case [1,6,7,10]. Angiography can identify and treat active arterial extravasation but cannot distinguish between healing mucosa and ongoing low-grade hemorrhage, a limitation noted in both trauma and gastrointestinal bleeding literature [22,23]. When recurrent bleeding persists without clear radiographic evidence, physicians face a therapeutic dilemma: to operate, as has been reported in cases where laparotomy confirmed mesenteric arterial bleeding and required segmental bowel resection, with variable outcomes, risking a non-therapeutic laparotomy, or to continue conservative care with ongoing uncertainty [24,25].

Capsule endoscopy offers distinct diagnostic advantages in such circumstances. Meta-analysis demonstrates yields exceeding 60% in obscure bleeding, significantly outperforming push enteroscopy and small bowel radiography [7]. In modern cohorts, capsule endoscopy achieved yields of 75% in overt bleeding and influenced management decisions directly [17]. Safety concerns about capsule retention have largely been resolved by the development of the patency capsule, which has been validated in both early and contemporary series [8,18]. Systematic reviews estimate overall retention rates near 2%, with substantially lower rates when patency testing or CT enterography is performed [19,20].

In the present case, capsule endoscopy performed on hospital day 36 confirmed healing of ileal ulcers without active bleeding or stricture, clarifying the source of recurrent hemorrhage at that time. These findings directly altered management by supporting conservative therapy and avoiding laparotomy in a frail, elderly patient, where surgery would likely have been pursued after recurrent hemorrhage despite embolization.

This case illustrates that capsule endoscopy, though traditionally a gastroenterology tool, can safely complement trauma care in selected patients. By providing direct mucosal visualization, capsule endoscopy filled a diagnostic gap left by CT and angiography, confirming that the ileal ulcers were healing rather than actively bleeding. This unique information directly supported the continuation of conservative management and helped avoid an unnecessary laparotomy. Although based on a single case, this report highlights capsule endoscopy as a potential diagnostic adjunct that can support conservative management and avoid unnecessary laparotomy in selected trauma patients. Broader generalization, however, requires validation in larger series or systematic studies.

## Conclusions

Delayed small bowel hemorrhage following blunt abdominal trauma is rare but clinically important. Conventional imaging and angiography, while indispensable in acute trauma care, often fail to fully characterize mucosal pathology in the subacute phase. Capsule endoscopy, preceded by patency capsule confirmation, can provide definitive diagnostic information in stable patients, guiding conservative management and avoiding unnecessary surgery. This case highlights the potential for capsule endoscopy to broaden diagnostic strategies and improve outcomes in trauma care.
